# New Appliances and Things Medical

**Published:** 1902-03-15

**Authors:** 


### NEW APPLIANCES AND THINGS MEDICAL.

[We shall be glad to receive, at our Office, 28 & 29 Southampton Street, Strand, London, W.C., from the manufacturers, specimens of all new preparations and appliances which may be brought out from time to time.]

### INVINCIBLE SOAP.

(Hodgson and Simpson, Calder Soap Works, Wakefield.)

Three samples of toilet soap manufactured by the above firm have been submitted to us for examination. Of these the Lion Scented Carbolic Soap we particularly commend to the notice of our readers, since it is among the few antiseptic soaps with which we are acquainted which combine the essential properties of a powerful germicide with the elegance of preparation which are demanded by the public in toilet specialities. Although there is incorporated in this soap an adequate percentage of carbolic acid, it is skilfully disguised with a very pleasant perfume which appears to cling to the hands after washing for quite as long as the antiseptic itself. We thoroughly recommend this germicide soap to the notice of surgeons and nurses, as well as to others whose occupations or vocations expose them to any form of infection. The two remaining soaps with which we are dealing are known as the Violet Toilet Soap and the Invincible Transparent Soap respectively. Both of these are very high quality toilet preparations, beautifully scented, economical in use, and as far as our examination demonstrates absolutely free from sugar or other deleterious ingredient.

### CATALOGUE OF INSTRUMENTS.

(Messrs. Krohne and Sesemann, 37 Duke Street, Manchester Square, London, W.)

The above catalogue of surgical instruments and apparatus will be thoroughly appreciated by medical men at a distance from town and by others who have neither time nor opportunity to keep themselves familiar with the rapid improvements which are daily being effected in the articles of manufacture for which Krohne and Sesemann are justly famous. The pages of this catalogue are profusely illustrated, and all goods are marked with a special reference number, with the price stated in clear figures. Apparatus for the treatment and concealment of physical deformities is a marked feature in Messrs. Krohne and Sesemann's list, and the ingenious contrivances which are specialities of the firm for the treatment of hammer toe, flat foot, bunions, etc., are well deserving of the attention of those who require orthopædic instruments.

